# Assessing the Relationship between Socio-demographic, Clinical Profile and Financial Toxicity: Evidence from Cancer Survivors in Sarawak

**DOI:** 10.31557/APJCP.2020.21.10.3077

**Published:** 2020-10

**Authors:** Shee-Ling Yap, Shirly Siew-Ling Wong, Keng-Sheng Chew, Jerome Swee-Hui Kueh, Ke-Lin Siew

**Affiliations:** 1 *Faculty of Economics and Business, Universiti Malaysia Sarawak, Kota Samarahan, Sarawak, Malaysia.*; 2 *Faculty of Medicine and Health Sciences, Universiti Malaysia Sarawak, Kota Samarahan, Sarawak, Malaysia. *

**Keywords:** Cancer, financial toxicity, socio-demographic, clinical profile, cancer survivors

## Abstract

**Background:**

Patient’s financial ability is always the most critical imputes to treatment choice and adherence; as it translates into health outcomes such as survival rate and quality of life. Cancer care is likely to affect the patient’s financial well-being, putting huge financial pressure to the families. Therefore, it is imperative to understand the confounding factors of financial toxicity among cancer survivors along the course of survivorship.

**Methods:**

This study was designed in the form of cross-sectional analysis, in which, cancer survivors were recruited from the Sarawak General Hospital, the largest tertiary and referral public hospital in Sarawak. To capture the financial toxicity of the cancer survivors, the Comprehensive Score for Financial Toxicity (COST) instrument in its validated form was adopted. Multivariable logistic regression analysis was applied to determine the relationship between financial toxicity (FT) and its predictors.

**Results:**

The median age of the 461 cancer survivors was 56 while the median score of COST was 22.0. Besides, finding from multivariable logistic regression revealed that low income households (OR: 6.893, 95% CI, 3.109-15.281) were susceptible to higher risk of financial toxicity, while elderly survivors above 50 years old reported a lower risk in financial toxicity. Also, survivors with secondary schooling (OR:0.240; 95%CI, 0.110-0.519) and above [College or university (OR: 0.242; 95% CI, 0.090-0.646)] suffer a lower risk of FT.

**Conclusion:**

Financial toxicity was found to be associated with survivors age, household income and educational level. In the context of cancer treatment within public health facility, younger survivors, households from B40 group and individual with educational attainment below the first level schooling in the Malaysian system of education are prone to greater financial toxicity. Therefore, it is crucial for healthcare policymakers and clinicians to deliberate the plausible risk of financial toxicity borne by the patient amidst the treatment process.

## Introduction

Cancer is a chronic disease that exhibits a huge global health concern. According to World Health Organization (2018), cancer accounts for 9.6 million global deaths. It is estimated that one in six deaths is due to cancer. Common cancers worldwide include lung, breast, colorectal, prostate and stomach cancers. In Malaysia, it is estimated that one in four Malaysian is suffering from cancer (Akhtari-Zavare et al., 2018).The five most common cancers include breast, colorectal, lung, lymphoma and nasopharyngeal cancer as reported in the Malaysian National Cancer Registry Report 2012-2016 (Azizah et al., 2019). In that period of time, there was 11.3% increase (or 103,507 new cases) in the incidence of cancer (Azizah et al., 2019). 

Besides that, out-of-pocket expenditure will cause the inequality of financial toxicity (FT) of cancer survivors (Zhu et al., 2020). FT describes the financial burden of cancer care faced by patients and their family members (Zafar et al., 2013). In this regard, cancer will lead to psychological, economic and financial consequences, including emotional impact on the quality of life, the need for financial adjustment, reduced workforce participation as well as bearing the cost of the cancer treatment and other treatment-related cost (Sharp et al., 2013). These financial burdens will cause some patients and their families to fall into debt or poverty, and imposed high level of distress along the treatment process (Kimma et al., 2012; Chang et al., 2013).

Past studies revealed that treatment-related financial burden is recognized as a barrier to the provision of high-quality care (Stump et al., 2013; Zafar et al., 2013). The greater the FT, the greater this adverse impact may be affecting the patient’s quality of life (Fenn et al., 2014; Chen et al., 2018; Park and Look, 2018).This may result in the patient having to forgo or delay his or her medical care (Kent et al., 2013) and to compromise his or her treatment adherence (Bestvina et al., 2014; Zafar et al., 2015). Carrera et al., (2018) similarly claims that FT is a major challenge faced by cancer patients. 

Whilst FT is an important issue, little is known about the predictors of FT (Fathollahzade et al., 2015; Bhoo-Pathy et al., 2019). Most prior studies that had been conducted focused on the association between financial distress and quality of life in developing countries (Chino et al., 2014; Lathan et al., 2016; Perrone et al., 2016; Chen et al., 2018; Park and Look, 2018). In Malaysia, the study conducted by Bhoo-Pathy et al., (2019) examined the factor associated with FT in various types of cancer, whereas Ting et al,. (2020) focused on urological cancer. 

Although past studies do provide insight on predictors on FT in Malaysia, the result may be differed due to different clinical profile, population demographic and healthcare services providers in Sarawak, Malaysia. The common cancers in Sarawak are breast, colorectal, lung and nasopharyngeal carcinoma or nasopharynx cancer (NPC). In fact, NPC cancer is one malignancy in Sarawak as it accounts for approximately 80 percent of NPC cases in Malaysia (Tiong and Selva, 2005). NPC is found predominantly among Chinese male, followed by the native Bidayuh ethnic group in East Malaysia. Therefore, this study attempts to address this research gap by evaluating the predictors of FT among cancer survivors in Sarawak. Besides that, we also examined the influence of socio-demographic and clinical differences on financial toxicity. 

## Materials and Methods

This study was designed in the form of cross-sectional research approach, in which, cancer survivors were recruited from the Sarawak General Hospital (SGH), the largest tertiary and referral public hospital in Sarawak. Specifically, face-to-face interview was conducted in the clinic of Radiotherapy and Oncology Unit (RTU) in SGH from February 2019 to June 2019. The minimum sample size required is 357 based on Sekaran and Bougie (2016) calculation, with 95 percent confidence level. To ensure adequate representative power of the sample size for each cancer type targeted in the present study, the distribution of required samples by cancer types was then computed using the proportion of cancer cases treated in SGH from 2014 to 2018. Only adult patients above 18 years old and diagnosed with either breast, colorectal, lung or NPC cancers will be recruited in this study. In this sense, purposive sampling was applied throughout the data collection process. Vulnerable groups such as prisoners, children, patients who are critically ills, patients with concomitant psychiatric illnesses, patients who are delirious or those without mental capacity (both temporarily or permanently) to adequately comprehend the contents of the participant information and intent of the study will be excluded. Furthermore, written informed consent will be obtained from the survey participants prior to the interview. On top of this, information on patient characteristics and treatment related profile such as cancer site, cancer stages, year after diagnosis and type of treatment were extracted from the medical records available in RTU. To assist the interview process with the participants with limited ability to convey in either English or Bahasa Malaysia languages, translators with high level of proficiency in some major local languages were recruited to overcome language barriers in data collection and minimize the potential biases due to miscommunication.

In this context, we applied the Twombly (2004)’s definition of cancer survivors, wherein the cancer survivor is defined as cancer patient who are survived upon the time of diagnosis and through the balance of their lives. The socio-demographic factors tested in the present study include patient’s age, highest educational attainment, ethnicity, area of residency and monthly household income. On the other hand, clinical profile of the patient encompasses of cancer site, cancer stage, year after diagnosis and type of treatments. The household income categories were computed based on the income group classification suggested by the Department of Statistics Malaysia (2017). In particular, household income were classified into bottom 40% (B40; < RM 3,000), middle 40% (M40; RM 3,000 - RM 6,275) and the top 20% (T20; > RM 6,275) of household earnings in Sarawak, using median as the central tendency measure. As part of the public health initiatives for population living under B40 income level to sustain the healthcare needs, B40 health scheme (PeKA B40) and mySalam health insurance scheme were introduced, apart from the existing financial assistances offered by local welfare departments as well as NGOs. 

*Instrument*

Financial toxicity was measured using the validated Comprehensive Score for Financial Toxicity (COST) instrument developed by De Souza et al., (2014). The COST instrument consists of 11 items measured on a 5-point Likert scale, with 0 indicates “not at all” and 4 indicates “very much”. The FT score ranges from 0 to 44, in which a lower score indicates a higher level of FT (De Souza et al., 2014). As there is no pre-defined threshold between the higher end and lower end of the FT scores, therefore, we extended the central tendency approach applied by several past studies (Huntington et al., 2015; Honda et al., 2018) to stratify the series of FT score into high and low risks of FT, on the basis of median. FT scores fall in the range beyond the median FT scores will be classified as “Low Risk of Financial Toxicity”, otherwise, “High Risk of Financial Toxicity” applies. 

*Ethical Consideration*

Ethical approval was obtained from the Medical Research Ethics Committee (MREC), Ministry of Health, Malaysia (NMRR-18-2686-44107). Furthermore, written consents to conduct data collection in RTU, SGH were obtained from the Director of Sarawak General Hospital as well as the Head and Consultant Medical Oncologist in the Department of Radiotherapy and Oncology, Sarawak. 

*Data Analysis*

The descriptive statistical analysis was utilized to summarizes the socio-demographic and clinical profile of the respondents to portray the composition and distributional patterns of the socio-demographic backgrounds and clinical features of the cancer survivors. In addition, univariate analysis was performed to observe how the socio-demographic and clinical features of the cancer patients influence the degree of financial toxicity as reported by the patients. Explicitly, the Mann-Whitney U and Kruskal Wallis tests have been conducted to develop the evidence of socio-demographic as well as clinical differences in the state of financial distress revealed by the cancer survivors. Alternatively, to model the relationship between financial toxicity with its confounding factors, multivariable logistic regression analysis was implemented with the stratified financial toxicity score computed upon the median financial toxicity score of 22.0. In this respects, odd ratios (OR), with 95% CI and p-value were tabulated for each independent variable, while Hosmer-Lemeshow goodness of fits statistics were enclosed to indicate the model fit. The research instrument used in the study was fully-adopted from the validated COST measurement scale developed by De Souza et al., (2014). Nevertheless, to reinforce the validity and reliability of the instrument in the local context of Sarawak, pilot testing was conducted prior to the main study. All items were tested for internal consistency using the Cronbach’s alpha test and variables were found to be reliable measures. All the statistical findings reported in this study were analyzed using the Statistical Package for Social Sciences (SPSS, version 25). 

## Results

In this study, 461 cancer survivors were interviewed. The median age of cancer survivors were 56 years (IQR=17.50). The majority aged less than 50 years old (31.2%) followed by 50 to 59 years old (28.2%) and 60 to 69 years old (28.9%). The largest group of cancer survivors were from Chinese ethnicity (41.0%) followed by Sarawak Indigenous group (35.4%) and Malay ethnicity (23.6%). Most of the cancer survivors (64.4%) was from income group bottom 40 percent (B40) household, 23.3 percent and 12.4 percent were from the income of middle 40 percent (M40) household and household income of top 20 percent (T20) household. In term of cancer stages, it is found 20.4% were in stage I, 26.0% were in stage II and stage IV and 27.5% was from cancer stage III. The summary of the respondents’ socio-demographic and clinical profile is presented in Table 1.

*The Inference of Socio-demographic and Clinical Differences on Financial Toxicity*

The median score of COST obtained in this study was 22.0 (IQR=14.00). As the data was not normally distributed, the variation between groups were evaluated with Mann-Whitney U and Kruskal Wallis tests (see Table 1). In the univariate analysis, cancer survivors showed older age, Chinese ethnicity, higher-income household, Kuching residence and those have a secondary school and above were associated higher level of COST score (less financial toxicity). In term of clinical profile, colorectal cancer survivors, early stage of cancer as well as survivors undergoes surgery have better financial well-being. However, there was no difference in length of cancer diagnose (p = 0.131) and survivors who received radiotherapy treatment (p =0.109) with financial toxicity. 

*The Multivariable Relationship between Financial Toxicity and Its Confounding Factors*

The findings of multivariable relationship between financial toxicity and its confounding factors were reported in Table 2. The overall logistic regression model was statistically significant, X^2 ^(20) = 123.335, p=0.000. There were 31.3 percent in the model can be explained based on Nagelkerke R square. The result of the Hosmer-Lemeshow test showed satisfactory goodness of fit for the model (p>0.05). Finding from the logistic regression revealed that age, monthly household income and educational level were significant predictors of financial toxicity. Remarkably, elderly above 50 years old were less likely to incur financial toxicity, compared to adults in younger age groups. In terms of education, survivors with at least secondary schooling (OR:0.240; 95%CI, 0.110-0.519) and better educated survivors who completed the college or university degrees (OR: 0.242; 95% CI, 0.090-0.646) show better financial well-being compared to those least educated group that acquires only the primary education or no formal schooling. Among others, household income is deemed to be the most critical aspect as survivors coming from low income household were up to 6 times more in suffering with financial toxicity (OR: 6.893, 95% CI, 3.109-15.281).

On the contrary, ethnicity, area of residency, cancer site, cancer stage and treatment options do not possess predictive content on financial toxicity. This finding implies that socio-demographic background plays a remarkable role in shaping the financial soundness of the cancer patients along the cancer survivorship. Instead, clinical features of the illness were not able to cofound the severity of financial hardship across the treatment process. In this regards, it is worth noting that socio-demographic elements, particularly the age, household income and education maintain some valuable inputs for better clinical decision making in cancer treatment, if patient’s risk of approaching into financial toxicity is to be accounted. Meanwhile, clinical profile of the illness could varies the financial commitment across the survivorship, but it reveals inconsequential role in driving the survivor into financial toxicity.

**Table 1:** The Inference of Socio-Demographic and Clinical Differences on Financial Toxicity

Variables	N	FT Score (≤22) (n= 242)	FT Score (>22) (n=219)	Univariate p-value
Age Group				
Less than 50	144	95 (39.3%)	49 (22.4%)	0.000^b^
50 to 59	130	70 (28.9%)	60 (27.4%)	
60 to 69	133	53 (21.9%)	80 (36.5%)	
70 and above	54	24 (9.9%)	30 (13.7%)	
Ethnicity				
Malay	109	63 (26.0%)	46 (21.0%)	0.000^b^
Chinese	189	74 (30.6%)	115 (52.5%)	
Sarawak Indigenous	163	105 (43.4%)	58 (26.5%)	
Area of Residency				
Capital City-Kuching	257	129 (53.3%)	128 (58.4%)	0.033^b^
Outside Capital City- Outside Kuching	204	113 (46.7%)	91 (41.6%)	
Education Level				
No Formal Education	62	46 (19.0%)	16 (7.3%)	0.000^b^
Primary Education	99	57 (23.6%)	42 (19.2%)	
Secondary Education	238	114 (47.1%)	124 (56.6%)	
College/ University	62	25 (10.3%)	37 (16.9%)	
Monthly Household Income				
Low-Income Household (B40)	297	195 (80.6%)	102 (46.6%)	0.000^b^
Middle-Income Household (M40)	107	36 (14.9%)	71 (32.4%)	
High-Income Household (T20)	57	11 (4.5%)	46 (21.0%)	
Primary Cancer Site				
Breast	174	88 (36.4%)	86 (39.3%)	0.003^b^
Colorectal	122	56 (23.1%)	66 (30.1%)	
Lung	77	43 (17.8%)	34 (15.5%)	
Nasopharynx	88	55 (22.7%)	33 (15.1%)	
Cancer Stages				
Stage I	94	46 (19.0%)	48 (21.9%)	0.002^b^
Stage II	120	55 (22.7%)	65 (29.7%)	
Stage III	127	62 (25.6%)	65 (29.7%)	
Stage IV	120	79 (32.6%)	41 (18.7%)	
Year after diagnosis				
Less than 1 year	97	49 (20.2%)	48 (21.9%)	0.131^b^
1 – 5 years	279	154 (63.6%)	125 (57.1%)	
5 years and above	85	39 (16.1%)	46 (21.0%)	
Chemotherapy				
Yes	335	187 (77.3%)	148 (67.6%)	0.019^a^
No	126	55 (22.7%)	71 (32.4%)	
Radiotherapy				
Yes	293	161 (66.5%)	132 (60.3%)	0.109^a^
No	168	81 (33.5%)	87 (39.7%)	
Surgery				
Yes	300	146 (60.3%)	154 (70.3%)	0.001^a^
No	161	96 (39.7%)	65 (29.7%)	

Lower FT score represents higher level or worse financial toxicity; COST, Comprehensive Score for Financial Toxicity; IQR, Interquartile range; ^a^, Mann Whitney P-value; ^b^, Kruskal Wallis P-value

**Table 2.** The Multivariable Relationship between Financial Toxicity and Its Confounding Factors

Factors	Odd Ratio (95%CI)
Age Group	
Less than 50	1
50 to 59	0.516 (0.292-0.911) †
60 to 69	0.189 (0.097-0.365) †
70 and above	0.168 (0.070-0.405) †
Ethnicity	
Malay	1
Chinese	1.678 (0.955-2.948)
Sarawak Indigenous	1.376 (0.792-2.394)
Area of Residency	
Capital City-Kuching	1
Outside Capital City- Outside Kuching	0.960 (0.614-1.503)
Education Level	
No Formal Education	1
Primary Education	0.538 (0.240-1.208)
Secondary Education	0.240 (0.110-0.519) †
College/ University	0.242 (0.090-0.646) †
Monthly Household Income	
High-Income Household (T20)	1
Middle-Income Household (M40)	2.207 (0.940-5.180)
Low-Income Household (B40)	6.893 (3.109-15.281) †
Primary Cancer Site	
Breast	1
Colorectal	1.514 (0.818-2.802)
Lung	1.641 (0.736-3.660)
Nasopharynx	1.336 (0.596-2.995)
Cancer Stages	
Stage I	1
Stage II	0.852 (0.440-1.648)
Stage III	0.844 (0.434-1.641)
Stage IV	1.415 (0.708-2.830)
Chemotherapy	
Yes	1
No	0.633 (0.371-1.079)
Surgery	
Yes	1
No	0.888 (0.476-1.658)

CI, Confidence interval; †, Statistically significant; Model coefficient X^2^(20), 123.335; p, 0.000; Hosmer- Lemeshow test (P=0.449); Nagelkerke R^2^, 0.313

## Discussion

Cancer-induced financial toxicity has become an important agenda in the provision of high quality cancer care. Findings from this study suggested that cancer patients from vulnerable income group and low educational attainment exhibit greater financial toxicity. This outcome is consistent with Honda et al., (2018) and Voit et al., (2019), in which disadvantage group in the community tends to suffer higher financial toxicity. Apart from that, Huang et al., (2019) maintained that cancer patients with lower educational attainment and household income are more likely to experience a higher risk of financial hardship. Therefore, providing a broader and more comprehensive healthcare protection scheme for these vulnerable groups is vital to cushion the financial impact of cancer treatment. In view of this, healthcare protection schemes aimed to improve the well-being and reduce the financial burden of those suffering from cancer need to be intensify to reach every corners of the rural and urban poor communities. 

To date, the Skim Peduli Kesihatan (more popularly known as “PeKa B40”), MySalam and MAKNA Bursary Assistance Programme were among the national protection schemes made available for these people since years ago. Unfortunately, little have benefited from these protection schemes, and this is especially true for the case of Sarawak given that none of these 461 cancer survivors recruited in this study has benefited from these schemes. In this sense, it is crucial for the government to have a closer look on the implementations of these schemes, as well as fostering the access to all these protection schemes into the wider community of Sarawak. Also, the available healthcare protection scheme is far beyond sufficient to cater the need of cancer treatment as prolonged treatment in cancer care is largely unavoidable, while the condition could be worsen by the rising healthcare cost. 

Moreover, patients who diagnosed with cancer at younger ages (50 years old below) reported greater financial toxicity than elderly survivors (above 50 years old). This is consistent with past studies such as Ramsey et al. (2013) and Kaddas et al. (2020). Younger cancer survivors are 2 to 5 times higher in bankruptcy rate and prone to greater financial toxicity compared to cancer patients aged 65 or older. This could be due to the fact that younger patients have more financial obligations compared to older adults as the latter group tends to have higher baseline household expenses and less accumulated assets (Shankaran et al., 2012). Besides, cancer treatment could disrupts job retention, leading to greater loss of potential earning and higher level of financial instability (Yabroff et al., 2016). Hence, the government as well as employment-related agencies are urged to provide adequate post-treatment support to these people, especially those working adults to resume their participation in job market through various platforms. This is extremely important for adults survivor who are also the head of household. Otherwise, cancer treatment is not merely stopping them from earning for daily needs, but also resulting in prolonged disruption in income stream to sustain the living needs of the family members. Ultimately, the well-being of the household will be slumped, and it possibly discourage patient’s treatment compliance. On top of this, patients with clearer insight and expectation of the treatment cost would be able to develop better financial planning to cope with the burdens of treating the illness as well as basic needs in the household. Individual’s ability to cushion the sudden financial distress may help to ease the fear of falling into financial toxicity, and therefore, strengthen treatment adherence among the patients with vulnerable socio-demographic backgrounds. 

All things considered, the present study also endures several limitations. Firstly, although heterogenous purposive samples collected and analyzed in this study could provide deeper insights into the understanding of financial toxicity among the cancer survivors in Sarawak. However, the clinical heterogeneity in terms of the types of cancer, stages of cancer and types of treatments are not well-segmented into subgroups analysis with a large set of data. Therefore, it is less desirable in providing a robust view for clinical decision support in each single type of cancer. Besides that, purposive sampling poses certain degree of biases due to its inability to randomize the recruitment process. Therefore, cancer survivors who are presence in the RTU clinic would not have equal chances of being selected as the survey participants. Moreover, patients who practice self-medication or complementary and alternative medicine were omitted as recruitment of survey participants only take place in the RTU clinic. This might infuses some selection bias into the study. Last but not least, the presence of response bias is also recognized despite researchers’ efforts to control the accuracy of the responses through a well-organized survey responding setting. 

Further studies may consider to extend the research into homogenous groups of cancer to provide better insights into the understanding of financial toxicity on a specific type of cancer. Secondly, cancer survivorship is a life-long journey, down the road, cancer survivors might face different kinds of late and/or long-term side effects of cancer treatment. Therefore, cross-sectional study can be further extended into a longitudinal exploratory study to capture the changes of financial toxicity at different phases of survivorship. In a nutshell, this study highlights the existence of financial toxicity among cancer survivors in Sarawak, even though public hospital is a highly subsidized healthcare provider in the State. In addition, this study also putting the vulnerable groups (younger age, low income, low education level) into central attention as these people are highly susceptible to financial distress amidst cancer treatment. In this respects, the policymakers and healthcare agencies are urged to enhance the financial protection strategies to ease the financial burden of the survivors from the vulnerable communities of Sarawak. Lastly, policy intervention on prevention and early detection of cancer could help to mitigate the risks of late diagnosis and delayed treatment. This is likely to bring down the cost of cancer treatment as well as securing a higher chance to survive from the illness. 
